# Lessons learned from the risk-informed urban development initiative in the SADC region

**DOI:** 10.4102/jamba.v16i2.1793

**Published:** 2024-11-22

**Authors:** Ketlaodirelang E. Letebele, Manuel A.A.L De Araujo, Johanes A. Belle, Frederika A. Shigwedha, Lucie N. Bakajika, Geofrey Ochieng, Georg Johann, Tlou D. Raphela, Jimmy P. Yoedsel, Gorata Samuel, Karl H.G. Sada

**Affiliations:** 1 South African Council for Planners, Development Planning, Environment and Management, eThekwini Municipality, Durban, South Africa; 2 Department of Climate Change and Energy, Quelimane Municipality, Quelimane, Mozambique; 3 Department of Disaster Management Training and Education Centre for Africa, Faculty of Natural and Agricultural Sciences, University of the Free State, Bloemfontein, South Africa; 4 Sub Division of National Government and Traditional Authorities Affairs, Ministry of Urban and Rural Development, Windhoek, Namibia; 5 Corporation of Congolese Town Planners, Kinshasa Multisector Development and Urban Resilience Project, Cellule Infrastructures, Kinshasa, DRC, Congo; 6 Department of Programme Management, East Africa Local Governments Association, Arusha, Tanzania; 7 Faculty of Hydrology & Hydraulics, Emschergenossenschaft & Flood Competence Center, Cologne, Germany; 8 Department of Energy, Environment & Climate Change, Asian Institute of Technology, Pathum Thani, Thailand; 9 Department of Environmental Science, Faculty of Science, University of Botswana, Gaborone, Botswana; 10 Global Initiative on Disaster Risk Management, Group Peace and Security, Deutsche Gesellschaft für Internationale Zusammenarbeit, Bonn, Germany

**Keywords:** risk-informed urban development, SADC, disaster risk management, resilience, risk-informed planning

## Abstract

This study presents the systemisation of lessons learned from the urban sector in which the measures based on the guiding principle of risk-informed development (RID) have been implemented in the Southern African Development Community (SADC) region at the national and/or sub-national levels. Despite notable risks in the region, these are not adequately considered in urban development planning and programming. Aiming at strengthening RID in the SADC region, the objectives of this peer-to-peer exchange were achieved through virtual workshops, roundtables and briefings on a cloud-based and open-source BigBlueButton Web conferencing system. Risk-based decision frameworks were used as footprints throughout the moderation phases. Peer-to-peer exchanges totalled 25 events and 80 registered individual members. About 14 SADC member states and 26 cities represented by city officials, local governments and academia participated in this process. Results along the risk-based decision frameworks lead to a clustering of recurring topics during the peer-to-peer exchange, which are presented along legal and organisational setups; budget and funding; risk assessment and mitigation options; and data and information flows considerations.

**Contribution:**

Aligned with the ‘Regional Assessment on Urban Vulnerability and Resilience in SADC Member States’ by conclusions provide a series of recommendations for risk-informed urban development in the SADC region.

## Introduction

### Background

The 2018 revision of the World Urbanization Prospects (United Nations 2019) anticipated the global urban population to increase by 2.5 billion urban dwellers by 2050 with about 90% of the increase concentrated in Asia and Africa. As part of sub-Saharan Africa which is experiencing an annual urban population growth rate of 4.1%, compared with a global rate of 2.0% (Saghir & Santoro 2018), the Southern African Development Community (SADC) member states are also urbanising at a fast rate (Simkins 2021). This exposes larger populations and infrastructure to the risk of disasters such as floods, droughts and cyclones that are exacerbated by climate change.

The SADC region encompasses 16 Southern African countries, housing a diverse consortium of vibrant market towns, rural communities, intermediate cities and metropolises, with tremendous opportunities for green economic growth and innovation (UN Habitat 2022). Despite this notable risk of disasters in the region, the changing land use and unsustainable urban development are not adequately considered in urban development planning and programming (Pourazar 2017). Climate change and extreme weather events in the SADC have adversely affected the region over the years and will become increasingly severe over the next few decades (Climate Change Adaptation in SADC 2011; Scholes & Engelbrecht 2021). The region’s exposure and vulnerability to disasters are inter alia linked to the river systems’ characteristics, its geomorphology, climate change and human-made activities, and these are exacerbated by factors such as unsustainable urban development (Rusca et al. 2023). Despite the increasing awareness of risk, these are still not adequately taken into consideration in development planning and programming. Current approaches usually address one threat at a time rather than considering several multiple threats and simultaneously occurring risks (Opitz-Stapleton et al. 2019). Disaster risk management (DRM) practices are still mostly concentrating on response to disasters rather than adopting cross-cutting and preventive approaches to minimising risk (Samuel et al. 2022). With limited resources to manage the threat of disasters or ensure the continuity of basic services provision of critical infrastructures and services, settlements are sites of concentrated risk, unabating vulnerabilities and repeated service delivery failures because of recurrent disaster impacts (Abbasabadi-Arab, Khankeh & Mosadeghrad 2022; Atanga & Tankpa 2022; Rusk et al. 2022). As such, reducing urban vulnerability, exposure and strengthening coping capacity and building resilience to a wide range of shocks in Southern Africa are central to ensuring sustainable and risk-informed urban development in the region.

Reducing urban vulnerability and ensuring sustainable urban development are shared priorities of several frameworks that this study was guided by and through the SADC Secretariat’s Disaster Risk Reduction (DRR) Unit under the SADC/GIZ Project ‘Global Initiative on Disaster Risk Management (GIDRM)’. Implemented by the Deutsche Gesellschaft fur Internationale Zusammernarbeit (GIZ), the GIDRM aims to build capacities and skills of selected decision-makers and regional organisations and initiatives globally, and specifically in Southern Africa, to enable context-specific use of risk-informed development (RID) guiding principles. The initiative on risk-informed urban development (RIUD) stems from the operationalisation of the implementation agreement between the SADC DRR Unit and GIZ from May 2022 till October 2023. Through the GIDRM, implemented by GIZ, [the SADC DRR Unit …], the SADC DRR Unit and in alliance with technical cooperation projects carried out, in partnership with SADC, measures for RID applied in the urban sector. In this sense, the initiative on ‘risk-informed urban development’ was carried out in cooperation with the Connective Cities – Community of Practice for Sustainable Urban Development (CC) and implemented by GIZ on behalf of the German Federal Ministry for Economic Cooperation and Development (BMZ). Global Initiative on Disaster Risk Management and CC addressed the complex nature of risks to safeguard development gains in the urban sector and the SADC region. This study presents the systematisation of lessons learned from the urban sector in which measures based on the guiding principles of RID have been implemented in the SADC at the national or subnational level.

Next to the operationalisation of the implementation agreement between the SADC DRR Unit and GIZ, RIUD aimed to strengthen and support agenda coherence among the different frameworks at global, continental and regional levels. For this purpose, specific consideration was given to the Sendai Framework for DRR 2015–2030 as the global footprint for DRR, which calls for the incorporation of DRR measures into multilateral and bilateral development assistance programmes within and across all sectors, as appropriate, related to […] urban development, and adaptation to climate change, as well as to address the complex nature of risks and safeguard sustainable development (United Nations 2015b). The Paris Agreement on the other hand:

[*E*]nhances the adaptive capacity, strengthening resilience and reducing vulnerability to climate change, […] ensuring an adequate adaptation response […] (Art. 7, §1); […] areas of cooperation […] may include: […] a) EWS […] e) CRA and CRM […] h) Resilience of communities, livelihoods and ecosystems (Art. 8 §4); […] strengthen […] cooperation on enhancing action on adaptation […] including […]: a) Sharing information, good practices, experiences and lessons learned […] relate to science, planning, policies and implementation in relation to adaptation actions; […] d) […] identifying effective adaptation practices, […] needs, priorities, […] challenges and gaps, in a manner consistent with encouraging good practices (Art 7 §7). (United Nations 2015a, pp. 9, 10 and 12)

The Sustainable Development Goals (SDGs) of the Agenda 2030 were also considered, specifically in their objective towards making cities and human settlements inclusive, safe, resilient and sustainable (SDG 11, target 11.5) (United Nations 2015c) and taking urgent action to combat climate change and its impacts (SDG 13) (United Nations 2015c). Further alignment for RID was considered as provided by the ‘New Urban Agenda Habitat III: Quito Implementation Plan for the New Urban Agenda’, which seeks to:

[*I*]ncorporate disaster risk reduction and climate change adaptation […] into […] urban and territorial development and planning processes […] while promoting cooperation and coordination across sectors and build the capacities of local authorities on DRR. (Item 101) (United Nations 2017, p. 25–26)

At the regional level, RIUD placed specific attention to the provisions made by the Agenda 2063 – The Africa We Want – to create an environmentally sustainable climate and resilient economics and communities (Aspiration 1, Goal 7) (African Union 2015). Within the SADC region, consideration of the SADC’s Regional Indicative Strategic Developmental Plan 2020–2030 (RISDP) pillars, namely, the ‘Infrastructure Development in Support of Regional Integration’ (Pillar 2); ‘Social and Human Capital Development’ (Pillar 3); and ‘Cross-Cutting Issues: Gender, Youth, Environment and Climate Change and Disaster Risk Management’ (SADC 2020), was given. Finally, and prior to the national and subnational levels. Priorities for the implementation of SADC Regional Resilience Framework 2020–2030 on ‘Robust and Connected Infrastructure’ (Priority 4) and ‘Sustainable Urban Centres’ (Priority 5), were considered and particular attention to strengthening the understanding of linkages between infrastructures, their interdependencies, […] as well as supporting the adoption of resilience in urban planning and integration of nature-based solutions (e.g. ecosystem-based DRR) into urban planning and development, were provided.

The joint initiative on RIUD therefore aims to facilitate:

[*T*]he development of scalable and/or replicable solutions while building up a network of cities and experts, addressing multi-actor, multilevel and cross-sectoral interdependencies, aiming at strengthening risk governance and risk-informed development. (SADC RIUD Report 2023)

The objective of this study is to present the peer-to-peer exchange on DRM for RIUD, which resulted from RIUD in the SADC region.

## Research methods and design

**FIGURE 1:**
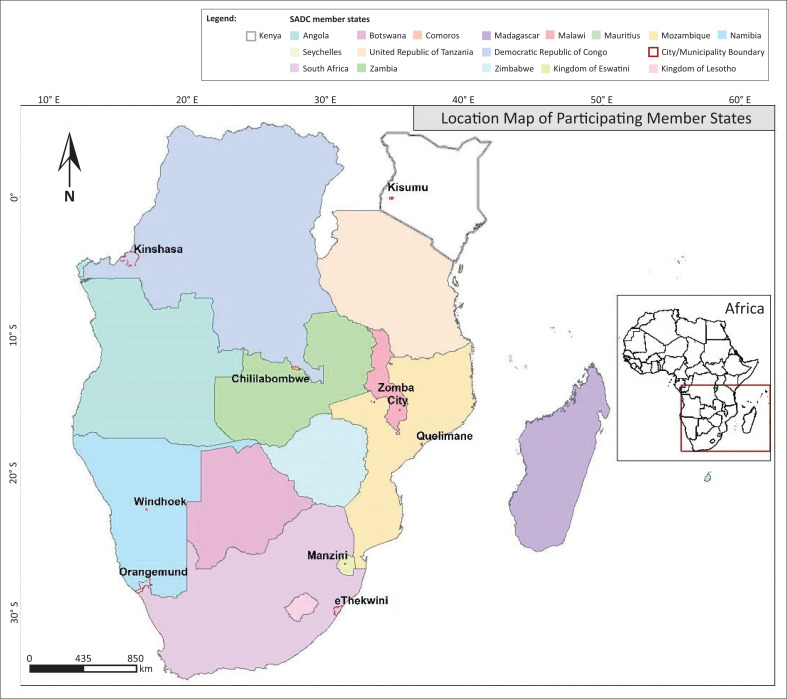
Map of the study area.

The inception of RIUD and its pilot phase commenced on 13 October 2021 during the International Day for DRR. Its implementation started in March 2022 and activities ended in October 2022, that is, 6 months of conceptualisation and inception, and 7 months of implementation. Risk-informed urban development pilot phase was organised along two main components, namely, ‘Working group on RIUD’ and ‘Insight moments on RIUD’ (SADC RIUD Report 2023). Figure 1 shows the location of participating member states, cities and/or municipalities in this study.

### Working group on risk-informed urban development

Group members of RIUD were invited first through the DRR-Focal Point List as indicated by the SADC-DRR Unit. Snowball sampling, as originated through the SADC DRR-Focal Points (see Table 1) and identification of potential working group on risk-informed urban development (WG-RIUD) participants, was complemented by those suggested by the City Climate Gap Fund mainly for the Southern African region, the CC GIZ programme for Germany as well as personal or professional networks provided by the coordinating group of RIUD (CC & GIDRM). Every potential WG-RIUD participant was addressed by email, and upon meeting agreement, a bilateral inception meeting was held. Following the expression of interest, a formal ‘Invitation to the Working Group on Risk-informed Urban Development’ was sent to each participant so that his/her participation and engagement could be confirmed. From March 2022 onwards, the WG-RIUD met regularly through virtual exchange formats (e.g. workshops, roundtables and briefings) hosted by the cloud-based and open-source BigBlueButton Web conferencing system under moderation. Moderation was informed by ‘Risk-based decision frameworks’, that is, International Risk Governance Council (IRGC) Risk Governance Framework (IRGC 2017); UK Climate Impacts Programme (UKCIP) Risk Framework, Foundations for Decision Making (UKCIP 2003); G20/OECD Disaster Risk Assessment and Risk Financing Methodological Framework (OECD 2012); ISO 31000:2018 Risk Management Guidelines; Swiss Agency for Development and Cooperation (SDC); and Climate, Environment, and Disaster Risk Reduction Integration Guidance (CEDRIG 2018) and structured along the common phases by Opitz-Stapleton et al. (2019). Moderation phases were thus within the frame of ‘(1) Scoping, (2) Risk Assessment/Appraisal/Screening, (3) Option appraisals and implementation, (4) Monitoring and evaluation, and (5) Communication phase’ (SADC RIUD Report 2023). Virtual exchange formats aimed to support and/or steer towards the formulation and submission of at least one ‘Expression of Interest’ for the City Climate Gap Fund.

**TABLE 1:** Contact points for the disaster risk reduction in Southern African Development Community (SADC) member states.

Variable	Country	Focal points to the SADC Secretariat, DRR Unit
1	Angola	Director, National Commission for Civil Protection
2	Botswana	Director, National Disaster Management Office, Ministry for Presidential Affairs Governance and Public Administration
3	Comoros	Deputy Director of Civil Security General Directorate of Civil Protection (DGSC) Ministry of Interior
4	Congo DR	Director of Civil Protection Department
5	Eswatini	National Disaster Management Authority (NDMA)
6	Lesotho	Disaster Management Authority
7	Madagascar	Executive Secretary, National Bureau for Disaster Risk Reduction
8	Malawi	Commissioner for Disaster Management Affairs (DODMA)
9	Mauritius	Director – National Disaster Risk Reduction and Management Centre at the Ministry of Local Government and Disaster Risk Management
10	Mozambique	President – Instituto Nacional de Gestão e Redução do Risco de Desastres (INGD)
11	Namibia	Directorate Disaster Risk Management, Office of the Prime Minister
12	Seychelles	Director General – Disaster Risk Management Division
13	South Africa	Head – National Disaster Management Centre
14	Tanzania	Director – Disaster Management Department, Office of the Prime Minister
15	Zambia	National Coordinator – Disaster Management and Mitigation Unit (DMMU), Office of the Vice-President
16	Zimbabwe	Director – Department of Civil Protection

*Source*: SADC RIUD Report, 2023, *Risk informed urban development: Lessons learned from the urban sector in which measures based on the guiding principle of RID have been implemented at the national and/or subnational level of SADC*, SADC Secretariat, Risk-Informed Urban Development | SADC, Gaborone

SADC, Southern Africa Development Community; DRR, Disaster Risk Reduction.

### Insight moments on risk-informed urban development along the United Nations Office for Disaster Risk Reduction-Making Cities Resilient 2030 Roadmap

The second component of RIUD’s initiative was the thematic inputs from different experts related to risk-informed development. Such inputs described as ‘Insight Moments’ were identified and organised during the moderation of WG-RIUD. Insight moments on risk-informed urban development (IM-RIUD) were open for all through the cloud-based and open-source BigBlueButton Web conferencing system (https://community.connective-cities.net/de/group/154/about). Following the United Nations Office for Disaster Risk Reduction (UNDRR) Making Cities Resilient 2030 (MCR2030) Roadmap to mainstream RID into decision-making, IM-RIUD was an open forum to all interested in cross-cutting topics like climate change adaptation, sustainable and resilient urban development. Insight moments on risk-informed urban development were held virtually, and upon agreement with the participants and keynote speakers, recordings were published on the CC community platform.

### Ethical considerations

This study does not contain any studies involving human participants performed by any of the authors.

## Results

As a joint venture between the GIDRM and the CC GIZ programmes, the RIUD initiative:

[*F*]acilitated the development of scalable and/or replicable solutions in the SADC region while building up a network of cities and experts, addressing multi-actor, multilevel and cross-sectoral interdependencies, aiming at strengthening risk governance and risk-informed development. (SADC RIUD Report 2023)

This result was realised by enabling peer-to-peer exchanges on DRR along the common phases of ‘Risk-based decision frameworks’:

- Scoping;
- Risk assesment, appraisal and screening;
- Options, appraisals and implementation;
- Monitoring and evaluation; and
- Communication phase.

The first section of the results, ‘WG-RIUD: Specific outcomes’, focuses on those from the common phases of ‘Risk-based decision frameworks’; while the second section, ‘IM-RIUD: Specific outcomes’, focuses on those from the IM-RIUD. The known yet unintended effects of the RIUD initiative are summarised in Table 4. Overall, peer-to-peer exchange (WG-RIUD and IM-RIUD sessions) totalled 25 events and 80 registered members under the cloud-based and open-source BigBlueButton.

### Working group on risk-informed urban development: Specific outcomes

The working group on RIUD was set up as a closed community of about 65 individuals (excluding GIZ staff and moderator), representing 14 countries and around 26 cities from the SADC region. It hosted a great and diverse group of city officials, representatives of local governments and/or municipalities from Southern Africa; members representing academia including the Disaster Management Training and Education Centre for Africa (DIMTEC) at the University of the Free State, South Africa; and the SADC’s DRR Unit Member State’s focal points at national level (see Table 2).

**TABLE 2:** Participants of the working group on risk-informed urban development section.

Number of participants	Number of countries	Number of cities	Target population
65	14	26	City officials, representatives of local governments and/or municipalities, academia (i.e. the Disaster Management Training and Education Centre for Africa [DIMTEC]), the Southern African Development Community (SADC) Disaster Risk Reduction Unit at the regional level and national level.

Working group on risk-informed urban development participants include representatives from Bonn (Germany); Chililabombwe (Zambia); eThekwini Municipality (South Africa); Kinshasa (DRC); Kisumu (Kenya); Manzini (Eswatini); Ministry of Housing and Urban and Rural Development (MURD, Namibia), Namibia; Quelimane (Mozambique); Windhoek (Namibia); Zomba (Malawi); academia and applied research from the DIMTEC Home (South Africa) and the Flood Competence Centre-Cologne (HKC) (Germany). The participants were encouraged to share, in an environment of trust and at a technical level, their own experiences, good practices, networks, tools, methods, legal frameworks, codes and/or financing mechanisms towards RID. The participants were also expected to identify roadmaps to guide decision-makers (including the working group [WG] participants) in understanding multiple threats, multiple and complex risks, and opportunities, as well as to identify the necessary steps for integrating knowledge of complex risks, tolerances, capacities, resources and policy priorities into development planning. Based on the guiding questions for each phase proposed by Opitz-Stapleton et al. (2019), results showcase the outcomes and peer-to-peer exchange along the existing risk-based decision frameworks. It is noteworthy that while the moderated sessions aimed to be contained within the limits of each of the decision frameworks, the WG-RIUD discussions as well as the reporting of the results showed multiple overlaps, which sometimes from the phase ‘Scoping’ touched, i.e., on a phase like ‘Communication and iteration’ and then to, that is, ‘Option appraisal’. Under this consideration, the results do not mirror the structure of the common risk-decision frameworks, but a cluster of recurring topics during the peer-to-peer exchange (see Table 3). As hotspots of exchange, topics may be understood as priorities, which in some cases refer to champions on RIUD while in others, address identified challenges for RIUD.

**TABLE 3:** Clustering of hotspots of exchange lessons learned along the common risk-based decision frameworks.

Thematic clustering	Key takeaways
Legal and organisational setups	Stakeholders tend to work in silos, leading to inefficiently utilised capacities and facilities for RIUD.
	Missing DRM frameworks.
	Traditional coordination mechanism.
Programmatic and actional setups	Constrictive understanding of mandates and competencies within the same organisation and institution beyond serves to close institutional gaps.
	Action plans can be the coordination basis for inter-sectoral departments.
Budget and funding	Budget and funding in the region are mainly focused on disasters.
	Disconnect between budgeting and allocation, between preparedness and response from international and national on DRM measures.
	Cities are often challenged to develop and implement preparedness plans on their own.
Risk assessment and mitigation options	Disaster risk assessment tools and frameworks that have been successfully piloted in the SADC region.
	Mitigation options for flood prevention were categorised by hard and soft engineering measures.
	‘Flood labelling initiative’ to inform urban dwellers in making informed choices when buying residential buildings.
Data and information flows	Scattered information sources and non-standardised data are a recurrent challenge in the region.
	International days can become leverage points for awareness raising and stakeholder engagement by showcasing the interlinkages between climate change adaptation, DRR and risk-informed development.

*Source*: SADC RIUD Report, 2023, *Risk informed urban development: Lessons learned from the urban sector in which measures based on the guiding principle of RID have been implemented at the national and/or subnational level of SADC*, SADC Secretariat, Risk-Informed Urban Development | SADC, Gaborone

RIUD, risk-informed urban development; DRM, disaster risk management; SADC, Southern African Development Community; DRR, disaster risk reduction.

### Insight moments on risk-informed urban development: Specific outcomes

Known as ‘Insight moments’ (IM-RIUD), thematic inputs from different experts were organised based on the needs identified during the moderation of WG-RIUD, yet open for all and offered to the wider global community. The open-access virtual sessions that took 60 min were, upon consent from the participants and keynote speakers, recorded and made available to the global community. Following the UNDRR-Making Cities Resilient 2030 (MCR2030) Roadmap, to mainstream RID into decision-making, ‘Insight moments’ complemented the expertise of the WG-RIUD by addressing capacity gaps and/or by presenting inspiring approaches for RIUD from your area of expertise and field of action (SADC RIUD Report 2023). The MCR2030 (link):

[*A*]ims to ensure cities become inclusive, safe, resilient and sustainable by 2030, contributing directly to the achievement of Sustainable Development Goal 11 (SDG11) ‘Make cities and human settlements inclusive, safe, resilient and sustainable’, and other global frameworks including the Sendai Framework for Disaster Risk Reduction, the Paris Agreement and the New Urban Agenda. (UNDRR 2024)

During March–11 July, IM-RIUD was held. The results here are structured according to the three MCR2030 Roadmap phases, namely, Stage A: Cities know better,^1^ Stage B: Cities plan better^2^ and Stage C: Cities implement better^3^ as follows:

#### Stage A: Cities know better focused on enhancing cities’ understanding on risk reduction and resilience

The United Nations Development Programme (UNDP) presented its RID approach focusing on securing development gains among multidimensional risks. The discussion was based on six focus areas that need interventions as follows: (1) evidence base (need for data analytics, risk assessments or profiling etc.); (2) policy (need to foster coherence, complementarities and co-benefits); (3) programmatic initiatives and implementation (need to institute a risk-sensitive development through capacity development, tools, methodologies and standards etc.); (4) engaging a diverse set of stakeholders (need for multi-stakeholder coalitions, ‘whole-of-society’ engagement, people-centred with focus on vulnerable, gender, at-risk, marginalised, etc.); (5) finance (need to mobilise public and private finance for investments in resilience, greater access to international funding streams etc.); and (6) knowledge and advocacy (need for cultural and behaviour shifts; creating ‘Champions’; political, administrative and societal leadership, etc.). The UNDP also highlighted the unmet demands and emerging priorities for urban resilience which among others were:

- diagnosing and addressing resilience attributes, inter-dependencies, and co-benefits;
- disconnected national policy to city application;
- political economy of urban development and decision-making; and
- weak accountability and resilience benchmarking.

Furthermore, the Asian Development Bank (ADB) showcased the lessons learnt and opportunities for pursuing RIUD where they highlighted that approaches for pursuing RIUD should not be business as usual but rather a bottom-up and decentralisation approach for (national) resilience plans, based on locally developed plans, thereby placing risk at the ‘centre’ of urban decision-making, instead of an ‘add-on’. Moreover, in terms of risk assessment, there is a need to:

- Know the type of development in which towns and which investments;
- Understand risk at systems level, instead of asset level (i.e. impacts because of interconnectedness between systems; transfer of risk from macro to micro level);
- Apply holistic resilience measures, i.e., ecological resilience (conservation/restoration of ecosystems, biodiversity use for resilience); physical resilience (climate/disaster risk-informed infrastructure); financial resilience (support financial preparedness in risk context); social and institutional resilience (pro-vulnerable, multi-faceted/scale resilience solutions);
- Apply integrated multisector and multiscale solutions;
- Adopt inclusive and locally led solutions (bottom-up initiatives);
- Invest in capacity building, i.e., ownership of local governments.

The following opportunities for pursuing RIUD were identified:

- Risk-sensitive land use management (informed growth and avoiding hazardous areas).
- Risk-informed social development (including social protection/health/education).
- Blue and green infrastructure (use nature-based solutions and other infrastructure for co-benefits).
- Hub for innovation (technology combined with inclusive processes).
- Innovative financing mechanisms (mobilise finance for resilience outcomes).

#### Stage B: Cities plan better, focused on improving assessment and diagnostic skills, increasing alignment between local strategies with national and regional strategies, and improving early-stage strategies and policies

Presentation led by the Joint Research Centre (JRC), UN Office for the Coordination of Humanitarian Affairs (UNOCHA) and UNDRR underlined the relevance of rigorous information systems as essential for risk-informed urban development in obtaining high-quality and real-time data. As the shifting risk attributes, patterns and manifestations are increasingly systemic, cascading and multidimensional with global, regional and transboundary impacts, the Risk INFORM tool can be used beyond risk assessment purposes, to engage with a diverse set of stakeholders, making RIUD people-centric and connect the baseline of risk information from regional, national to local policy and decision-making processes.

The German Aerospace Centre (DLR) also gave a presentation on multi-risk exposure modelling using earth observation techniques for natural hazard risk assessment where they shared different methodologies to multi-risk analysis. Scenario-based multi-risk assessment in the Andes region (RIESGOS) approach was one of the approaches under discussion. In summary, RIESGOS approach is based on:

- multi-risk scenarios (earthquake and tsunami events and their impact on residential buildings and critical infrastructure);
- dynamical analysis of cascading processes (based on users’ selection of parameters); and
- independent and distributed web services (connected through a web platform (demonstrator) that serves as the interface for user interaction and visualisation of results).

According to the Asian Institute of Technology (AIT), there is a growing need to improve regional climate modelling, as the impacts of climate change are felt at the local scale, but data are mostly available at the global scale. As lack of data at sub-national scales often stands in the way of carrying out local climate-related assessments, downscaling has been proven to be one of the most effective methods for receiving necessary data. This applies to all cross-cutting topics that determine a city’s resilience.

In terms of exposure modelling, that is, automated characterisation of exposed buildings with street-level imagery, the German Aerospace Centre (DLR) indicated that the exposure of cities stands in high dependence on their different building types. Therefore, knowledge of the structural properties of buildings exposed to natural hazards is critical for creating updated vulnerability maps and accurate risk models and for defining disaster management strategies. Geotagged street-level imagery yields a high potential for the automated inference of vulnerability-related building characteristics. The advantage of using such methods is its accurate large-area derivation of on-site information and its automatisation, which guarantees high efficiency.

Technical University of Munich (TUM) highlighted that given the interdependencies of natural hazards and risks, it is critical to carry out vulnerability and resilience assessments in cities that consider the impacts and effects between them. Because of their vital role in urban societies, critical infrastructures hold a high rank in terms of risk prevention and reduction. Infrastructure networks are already very intertwined among themselves and trigger multiple complex threats, especially when multiple hazards occur. The presented approach can be used to achieve more detailed vulnerability assessments that specify these cascading effects and help prepare RID.

The German Research Centre for Geosciences (GFZ) further discussed that, vulnerability is a key concept within DRM and is often the first step towards the planning of any disaster mitigating or climate adaptation measures. With rising multi-hazard scenarios in cities, it is important to fully understand the dynamics of vulnerability and to find ways to quantify it. The presented approach contributes to create a decentralised and inter-operative system for the exploration of multi-hazard risk scenarios, by using an exposure framework that harmonises existing models. It therefore delivers a big potential for improved risk assessment in a multi-hazard context of cities, which can be communicated to urban decision-makers.

#### Stage C: Cities implement better, focused on supporting cities in the implementation of risk reduction and resilience actions

For cities to implement better, Cologne University of Applied Sciences (THK) mentioned that it is important to understand the concepts of risk, vulnerability and resilience regarding critical infrastructures as well as the possibilities for building capacities on a local level, for ensuring holistic RIUD approaches. While technological advances accelerate interconnectivity within cities, increasing disaster risks occur with unprecedented intensity and frequency, which leads to even higher damages and further transmitted impacts. Because of their vital role in urban societies, critical infrastructures hold a high rank in terms of risk prevention and reduction and must be protected.

On the other hand, the Disaster Risk Management Sustainability and Urban Resilience (DiMSUR) introduced the City Resilience Action Planning Tool (CityRAP-Tool) in which they indicated that increasing urban problems faced by intermediate and secondary cities because of urbanisation, climate change and lack of rigorous planning guidelines require sustainable solutions that are pursued across departments and stakeholders. One way to achieve this is through the CityRAP Tool. It is an inclusive, participatory resilience planning method that can create a participatory approach between communities, practitioners and local leadership. Complementary to this, the City Climate Finance Gap Fund (Gap Fund) provides technical assistance for climate-smart urban planning and investment in low- and middle-income countries.

The United Nations Office for Disaster Risk Reduction concluded with a presentation on the MCR2030 Dashboard and Disaster Resilience Scorecard. The MCR2030 is a cross-stakeholder initiative aimed at improving local resilience through advocacy, knowledge sharing and experiences while establishing and reinforcing city-to-city learning networks by injecting technical expertise, connecting multiple layers of government and building partnerships (SADC RIUD Report 2023; UNDRR 2024). Its programmatic, flexible and iterative approach is built around a clear three-stage ‘resilience roadmap’ that supports cities on their journey to reduce risk and build resilience. Making Cities Resilient 2030 contributes directly to the achievement of SDG11 ‘Make cities and human settlements inclusive, safe, resilient and sustainable’ and other global frameworks including the Sendai Framework for DRR, the Paris Agreement and the New Urban Agenda (Table 4).

**TABLE 4:** Spin-off and unintended effects from risk-informed urban development.

Effect or Spin-off from RIUD	Description
The G7 – Ministerial Meeting on Sustainable Urban Development – Informing G7/U7: Nature-based-solutions for risk-informed urban development planning and sustainable infrastructure	Recognised its common goal to maintain and improve the quality of life in cities of all sizes in their pursuit of urban resilience and sustainable transformation.
	Recommended to strengthen cities’ capacities to monitor crises at an early stage and increase the integration of risk and crisis management as part of a preventive urban development policy to create urban resilience.
	Committed to joint action by continuing and aligning existing alliances and establishing new initiatives, particularly on the joint development of and exchange of strategies to increase urban resilience to prevent, protect against and adapt to imminent crises and disasters threatening livelihoods on a global scale.
National Level – Risk Inform Subnational Level – Eswatini	The Eswatini National Disaster Management Agency (NDMA) approached GIDRM in the search for assistance and guidance on options for adopting the ‘Risk Inform Sub-national level’ for application in Eswatini.
	Developing an SADC Risk Inform Sub-national level equivalent to one of the Sahel regions funded by ECHO would be seen as ideal as preparedness as well as prevention could be informed from a regional perspective with sub-national detail.
	Suggested a pilot exercise including training for developing the Risk Inform Sub-national level model in Eswatini and other SADC MS could as a regional approach be more strenuous on financial and time resources.
City Level – Bonn to become MCR2030 Resilience Hub	Making Cities Resilient 2030: Unleashing the potential of networking in disaster prevention – Bonn as a future resilience hub” announced […] that it intends to apply as a resilience hub until the end of the year (2022).
Research – Master’s Thesis	‘Harmonizing climate change adaptation and disaster risk management through risk informed development’ by Mr. Jimmy Yoedsel, at the Masters in Climate Change and Sustainable Development from the Asian Institute of Technology, Thailand.
International exposure of RIUD – 7th World Congress and Summit of Local and Regional Leaders	The session ‘Flood Risk Management and the Importance of Municipal Preparedness’ was organised by Connective Cities, the Association of German Cities (DST) and Engagement Global gGmbH/Service Agency Communities in One World (SKEW) in collaboration with the GIDRM, GIZ.
	The session objective was to enable participants’ insights into good practice examples in the field of flood prevention, protection and management and to create a common learning and exchange process.

*Source:* SADC RIUD Report, 2023, *Risk informed urban development: Lessons learned from the urban sector in which measures based on the guiding principle of RID have been implemented at the national and/or subnational level of SADC*, SADC Secretariat, Risk-Informed Urban Development | SADC, Gaborone.

RIUD, risk-informed urban development; GIDRM, Global Initiative on Disaster Risk Management; SADC, Southern African Development Community; MCR, Making Cities Resilient; GIZ, Gesellschaft fur Internationale Zusammernarbeit; ECHO, European Civil Protection and Humanitarian Aid Operations; MS, Member States.

## Discussion

Southern African Development Community member states are largely affected by flooding which eventually led to disruption of daily activities and even fatalities. Literature supports the claim made by the WG-RIUD that tropical cyclones are the worst-ever natural hazard to affect Southern Africa (Bopape et al. 2021; Dube, Chapungu & Fitchett 2021; Mutasa 2022), and these are principally influenced by climate change. Furthermore, Samuel et al. (2022) indicated that:

[*F*]lood disasters come about due to intersection of flood waters with areas that are unprepared to deal with flooding, mostly due to poor physical planning and layout of developments and corresponding storm drainage networks especially in urban areas.

Hence, the need for an RIUD.

It is evident that there are plenty of frameworks, strategies and action plans guiding DRM but from the WG-RIUD and IM-RUID results, stakeholders have reported that working on the same objective without proper communication makes available capacities and facilities underutilised. Because of inefficiencies, hazards often turn into disasters, urging thus to break silos for better coordination and/or mandate’s articulation. Atanga (2020) validated this assertion as they revealed that flood prone community leaders only participate during the implementation stage of flood risk management strategies. However, in order to have an effective flood risk management strategy, they have to take part in every stage of its development.

Despite the limited or lack of participation of stakeholders in DRM, the peer-to-peer exchange established that there is also a lack of DRM frameworks that provide coordination guidance which indirectly increases the probability of hazards turning into multiple ones and finally into disasters. In the attempt to address this, research institutions and/or universities assume the role of strengthening policy-research interface by providing rigorous grounds for decision-making where there seem to be institutional vacuums and/or missing DRM frameworks.

Another noted mechanism relating to missing and/or weak coordination schemes is multilevel governance between regional – national – provincial – municipal and/or sectoral across levels, where consensus and/or interfaces cannot be recognised. Croese et al. (2021), Ishtiaque et al. (2021) and Maldonado, Maitland and Tapia (2010) added that the multi-organisational nature of disaster response is challenging, especially in information systems development, but there still has been limited attention given to the structuring of relations and their associated power dynamics at different levels. Results further indicated that for better multilevel governance purposes, international cooperating or direct implementing partners can play a central role for convening, moderating and mobilising multiple stakeholders. This is supported by Croese et al. (2021) that a strong framework for multi-stakeholder engagement and coordination at all levels of governance is required through the adoption of both top-down and bottom-up approaches, used simultaneously.

The results of this study further highlighted the relevance of a ‘traditional coordination mechanism’, which has a stronger focus on preparedness. This includes the local inhabitants, ‘civil protection committees’, the Red Cross, the Fire brigade, and Police as examples of institutionalised schemes. This shows a combination of endogenous and exogenous coordination mechanisms as well as top-down and bottom-up approaches. Examples of improved coordination mechanisms were identified in Namibia, where the interface between the Directorate Disaster Risk Management at the Prime Minister’s Office and the Ministry of Urban and Rural Development actively unpacked the ‘Disaster Management Act Num. 10 of 2012’ (SADC RIUD Report 2023).

Another example was derived from eThekwini (South Africa), which was particularly interesting as agents of change, who actively addressed the interconnectedness at a horizontal level. Still on eThekwini, results point out that action plans can facilitate coordination for inter-sectoral departments, for instance:

[*T*]he ‘Durban Climate Action Plan’ which calls and empowers the participation of departments like ‘Disaster Management’, ‘Human Settlements’, ‘Area-Based Management’, ‘Environmental Planning and Climate Protection’, ‘Engineering Unit’, ‘Parks, Recreation and Culture’ and ‘Spatial Planning’ Departments’ across sector boundaries. (SADC RIUD Report 2023)

At the project level, results further identified the ‘Transformative riverine management program (TRMP)’, which was initiated by the water sector in eThekwini as a vehicle for holistic flood preparedness and risk reduction in informal settlements as well as configuring an early warning system tailored to facilitate communication between settlement dwellers and trained response team leaders from the disaster management control room.

In terms of budgeting and finance, results established that budget and funding in the region are still mainly focused on disasters at both national and international levels. Some SADC member states have disaster funds in place, that is, for tents and food, which means they mainly focus on response and/or providing relief. However, while DRM is budgeted at the national level and mainly managed at the national level, competition for allocation at the subnational level for any DRM matters was observed. Moreover, Nemakonde and Van Niekerk (2022) established that both DRR and Climate-change adaptation (CCA) are underfunded but much of the funding for DRM is allocated for recovery activities and less on preparedness and risk reduction activities by the SADC member states:

[*W*]ith a disconnect between budgeting and allocation, between preparedness and response from international and national on DRM measures, cities are often challenged to develop and implement preparedness plans on their own and through other budget items. (SADC RIUD Report 2023).

In other words, an assumption is made by the municipalities over prevention, for example, Manzini (Eswatini) developed hazard maps to inform vulnerability reduction measures. Moreover, endogenous preventive and reactive committees in DRC are the most effective in comparison with the nationally organised committees (SADC RIUD Report 2023).

Results additionally showcased some of the disaster risk assessment tools and frameworks that have been successfully piloted in the SADC region, that is, the ‘Community-based resilience analysis’ (CoBRA), ‘Technical Assistance to Non-Governmental Organizations’ (TANGO) and the ‘Community Capital Framework’. During these assessments, it was emphasised that risks need to be assessed as a complex system, in which behaviour and decision-making in the network determine the exposure and vulnerability. Through awareness of the systemic nature of hazards, mitigation options for flood prevention were categorised by hard and soft engineering measures:

Hard measures refer to i.e. dikes and flood retention walls, etc.; while soft: included the amending current land use regulations, flood insurance, raising awareness and education of citizens to flood risk, etc. (SADC RIUD Report 2023)

Specific examples from the results included strengthening building codes and regulations, comprehensive river management, reducing deforestation and land use changes while restoring native land covers. ‘Flood label’ adapted and proven measure used by Ghana to assess the flood risk in residential properties was provided as a recommendation to reduce flood risk and allow residential owners to engage in taking necessary measures to improve their ‘label value’. The ‘Flood labelling initiative’ on the other hand highlighted opportunities on how to inform urban occupants in making informed decisions when purchasing residential buildings as well as encouraging the housing market to develop less exposed and vulnerable buildings (SADC RIUD Report 2023).

In terms of data inflow, scattered information sources and non-standardised data were identified as a recurrent challenge in the region. Results showed that when capacities upon disasters are limited, the media becomes indirectly the verification source for further decision-making. While this doesn’t imply a critic bust suggests a window of opportunity, early warning systems and risk data (by involving the Meteorological Services and Departments for Early warning system (EWS) development and communication) were also described as favouring domestic and international investment. Results further suggested the use of international days for awareness-raising and stakeholder engagement, precisely by showcasing the interlinkages between climate change adaptation, DRR and RID. As a means of communication, beyond the written text, art was also noted to transcend communication barriers (technical language, culture, geography, age and background) (SADC RIUD Report 2023).

## Conclusion

Risk-informed urban development working group members gave their inputs and concluded that singular risks such as flooding affect various other sectors within municipalities/provinces, and these are created by our (sectoral) development decisions that are increasingly interconnected and systemic. A well-coordinated and collaborative approach must be taken to transform current risk governance towards RIUD. Inclusive approaches to risk governance support the involvement of the government across national, regional and municipal scales working together with academia, news media, private businesses, NGOs and civil society. By ensuring a role by all those affected by risks giving such actors a responsibility in handling their own resilience, vulnerability inherent in the city would be systematically addressed, thus leaving no one behind.

Cities have become the centre of human activity and thus are exposed to the confluence of risks and vulnerable groups that are mainly found in the informal settlements and disproportionately affected are women and children:

Challenges to the well-functioning of infrastructure in cities such as invasive plant species can also be turned into a job-creation opportunity by securing the sustained functioning of critical infrastructure to cities and its networks. The sustained functionality of cities also requires coordination beyond boundaries and a systemic view to keep such functionality by intertwining climate change adaptation and DRR at multiple scales and the working-group on RIUD, as well as other initiatives are relevant for ensuring that development gains can be sustained. (SADC RIUD Report 2023)

For instance, the Urban Risk and Management and Resilience Strategy by the UNDP aims to expand the list of cities in partnership and in cooperation with MCR2030, to an additional 30 cities where the concept of RIUD could be a key component to the strategies’ implementation approach. Another good example of RID is the ‘Transformative Riverine Management Programme’ from eThekwini. Lastly, a city-to-city exchange facilitated by GIZ demonstrated innovation on both cooperating parts while fostering unfolding synergies with other cities and co-financing sources (SADC RIUD Report 2023).

The following conclusions and/or recommendations are articulated according to the structure of recommendations provided by the ‘Regional Assessment on Urban Vulnerability and Resilience in Southern African Development Community Member States Strengthening Capacities for Reducing Urban Vulnerability and Building Resilience in Southern Africa’ (UN Habitat 2022) and drawn from this report as follows:

### Enhancing policy, legislation, institutional and organisational setups with a stronger risk-informed development focus

Risks are created by our (sectoral) development decisions and are no longer a standalone matter but rather increasingly interconnected and more and more systemic, hence the integration of climate change adaptation. The study therefore recommends that investment in evidence-based decision-making processes is essential to:

(1) [*E*]nsure that risks are integrated into development decisions; (2) ensure that the kind of policies that are made are also risk-informed; and (3) ensure that risk-informed development informs choices and decision at all levels, and is people centered. This will therefore strengthen decision makers (including the national and local parliaments) to include risk informed development. (SADC RIUD Report 2023)

The study further suggests recalibration of the international architecture of International Cooperating Partner (ICPs) for better integrated and efficiently financed climate change adaptation and DRR. A separate policy on CCA and DRR is not needed, but rather one that is directed to city resilience plans and the integration of both approaches (SADC RIUD Report 2023).

### Invest in capacity building, knowledge and information management

The study concludes that existing tools for DRR-CCA mainstreaming can be used and have been already piloted, what remains is to ensure that multiple actors have the tools and processes known to them and are not exclusive to administrative entities. The study suggests that there is a need to raise awareness of risks (hazards, vulnerability, exposure) among the population through training and/or capacity building to reduce vulnerability and exposure among the local population while educating with contents that promote adaptation and climate action. The study also recommends information and knowledge sharing among hazard mapping by communities as significant elements for disaster preparedness strategies in cities and local governments while also capitalising on data production and availability for better communication, thus proving decision-making processes for RIUD (SADC RIUD Report 2023).

### Strengthen regional and national urban planning for building resilience

The study concludes that cities have become the centres of human activity and thus are exposed to the confluence of risks; therefore, RIUD is so relevant. The study further indicates that vulnerability mapping requires interdisciplinary teams that imply the involvement of different departments and services for option appraisals. The study thus recommends the need to strengthen local governments as first in authority during disaster preparedness as they plan with the community while ensuring greater synergies. Moreover, it is imperative that development interventions and RID approaches are centred in African cities, especially the youth, as many cities are emerging, transitioning, intermediary and/or border towns (SADC RIUD Report 2023). The call for interventions on RID is now.

### Disaster risk financing and socio-economic considerations

Private and/or individual developmental decisions through capacity building equip citizens with options and awareness on how RIUD ensures the economic survival of cities while considering gender equality, different vulnerability groups (in the informal settlements, women and children, etc.) and different adaptation measures. The study further established that gender roles differ from one society to another and acknowledged that communities associate high-risk exposed areas with their way of life. Therefore, to reduce their developmental risks in cities, developmental gains options and socio-economic norms need to be changed. The study indicates the need for attention to different groups, roles and powers of vulnerability of different stakeholders as urban residents are affected by project implementation differently. The study recommends that cities should climate-proof all their projects to mainstream RID, thereby paying attention to urban dwellers’ and citizen’s knowledge, particularly in capitalising on the know-how of the technical staff at the local governments for better risk governance. It is also proposed that cities should ‘budget(ize) risk’ – like in human health and sickness, prevention is more cost-efficient for ensuring health and developmental gains (SADC RIUD Report 2023).

### Offer and promote durable solutions

The study concludes that the interdependencies between different sectors, that is, solid waste management, drainage and health are heightened through regular maintenance and repairs because of different reasons in infrastructure. Therefore, the promotion of locally available materials for reducing dependencies and linking RIUD to local economic development is suggested by the study. Moreover, there is a need to utilise nature-based solutions for climate change adaptation measures and preparedness (SADC RIUD Report 2023).

### Strengthen multi-level, inter-country and inter-city cooperation

The conclusion derived from this study is that the consistent functionality of cities requires proper coordination beyond boundaries and a systemic view to keep such functionality by linking climate change adaptation and DRR at multiple scales. Furthermore, the working group on RIUD and other initiatives are relevant for ensuring that development gains towards the Agenda 2030 can be sustained. The Urban Risk and Management and Resilience Strategy by UNDP on the other hand intends to increase the number of cities in cooperation with MCR2030 where RIUD is a key component (SADC RIUD Report 2023).

The city-to-city climate partnerships should entail concepts of RIUD. Such visioning processes should be carried out jointly between city-to-city partners from the inception of every partnership at the local government. It is therefore recommended that:

[*C*]ommon grounds should be found to overcome barriers of communication and consultation between national and local governments when building (social) and critical infrastructure. Challenges to the well-functioning of infrastructure i.e. invasive plant species can be turned into a job-creation opportunity by securing the sustained functioning of critical infrastructure to cities and its networks. (SADC RIUD Report 2023)

## Footnotes

1 Resilience Roadmap: Resilience Roadmap Stage A | Making Cities Resilient 2030 (undrr.org)

2 Resilience Roadmap: Resilience Roadmap Stage B | Making Cities Resilient 2030 (undrr.org)

3 Resilience Roadmap: Resilience Roadmap Stage C | Making Cities Resilient 2030 (undrr.org)
